# The association between the serum uric acid to creatinine ratio and all-cause mortality in elderly hemodialysis patients

**DOI:** 10.1186/s12882-022-02798-4

**Published:** 2022-05-06

**Authors:** Zhihui Ding, Yao Fan, Chunlei Yao, Liubao Gu

**Affiliations:** 1 Department of Nephrology, Taizhou Second People’s Hospital, Taizhou, China; 2 Division of Clinical Epidemiology, Affiliated Geriatric Hospital of Nanjing Medical University, Nanjing, China

**Keywords:** SUA/Scr, Hemodialysis, Elderly, All-cause mortality

## Abstract

**Background:**

Elderly hemodialysis patients have a higher rate of mortality than nonelderly hemodialysis patients. Recent studies shown that the serum uric acid to creatinine ratio (SUA/Scr) was associated with all-cause mortality in general adults. The purpose of the present study was to investigate the association between the SUA/Scr and all-cause and cardiovascular disease mortality among elderly hemodialysis patients.

**Methods:**

A total of 222 patients (≥ 60 years) who received hemodialysis more than 8 h per week at Taizhou Second People’s Hospital for at least 3 months were enrolled in the present study from January 2015 to December 2019. Clinical characteristics including age, sex and height et. al, were obtained from the hemodialysis database. The laboratory data, including albumin (ALB), total cholesterol (TC), serum uric acid (SUA), serum creatinine (Scr) and so on, were collected before hemodialysis and analyzed by automatic biochemical analyzer. Survival information was recorded during the follow-up period. Multiple Cox regression was carried out to analyze the association between SUA/Scr and all-cause mortality. The survival rate of each group was calculated by the Kaplan–Meier method, and the ratio of survival curves was analyzed by the log-rank test. The contribution of SUA/Scr for predicting all-cause mortality risk was evaluated by net reclassification improvement (NRI).

**Results:**

During the 19-month observation period, 78 patients died. Individuals in the nonsurviving group had significantly older ages (*P* < 0.001), body mass index (BMI) (*P* = 0.004), serum creatinine (*P* = 0.005) and prealbumin (*P* = 0.006) than surviving patients. After adjusting for age, sex, BMI, prealbumin, dialysis vintage, dialysis frequency, single-pool Kt/V (spKt/V), DM, hypertension and comorbidities, a higher ratio of SUA/Scr was independently associated with a higher risk of all-cause mortality (HR: 1.292; 95% CI: 1.013–1.648; *P* = 0.039). The predict value on all-cause mortality of SUA/Scr was superior to SUA (additive NRI = 0.214, *P* = 0.015) and Scr (additive NRI = 0.476, *P* < 0.001) among elderly hemodialysis patients.

**Conclusion:**

The serum uric acid to creatinine ratio is strongly associated with all-cause mortality in elderly hemodialysis patients which is more predictive than SUA or Scr alone.

**Supplementary Information:**

The online version contains supplementary material available at 10.1186/s12882-022-02798-4.

## Introduction

According to the 2019 United States Renal Data System (USRDS) annual data report [1], the crude incidence rate of end-stage renal disease (ESRD) was 370.2 per million per year in the United States, with 86.9% of ESRD patients receiving hemodialysis treatment. Currently, hemodialysis is the main renal replacement therapy among elderly ESRD patients. However, elderly hemodialysis patients still had a three- to six-fold higher mortality risk than nonelderly hemodialysis patients in the Dialysis Outcomes and Practice Patterns Study **(**DOPPS) study [2]. There are many identified factors contributing to the increased mortality risk in these patients, such as blood pressure, urine albumin, anemia, and serum creatinine [3–8].

Among these multiple risk factors increasing the mortality risk of hemodialysis patients, serum uric acid (SUA) has recently captured much interest and has been considered as a new member. It has been extensively shown that the SUA level is positively correlated with all-cause mortality [9–11]. However, the actual association between SUA and all-cause mortality in hemodialysis patients remains unclear and controversial. For example, Zawada AM *et al.* revealed that SUA level and all-cause mortality showed a U-shaped pattern among hemodialysis patients [12]. Thus, more data are needed to clarify this issue.

Previous studies demonstrated that the renal function-normalized SUA (SUA to creatinine ratio, abbreviated SUA/Scr) was more sensitive than SUA in relation to Parkinson’s disease, metabolic syndrome (MetS), nonalcoholic fatty liver disease (NAFLD) and chronic obstructive pulmonary disease (COPD) [13–20]. We also found that SUA/Scr could predict renal disease progression (incident CKD and ESRD) and correlate with β-cell function among type 2 diabetes patients [21–23]. A recent study from a US national survey showed that a higher ratio of SUA/Scr was associated with increased all-cause mortality among adults [24].

To date, the relationship between SUA/Scr and mortality risk among hemodialysis patients has not been reported, possibly because that Scr level could not reflect the renal function precisely in chronic hemodialysis patients, especially in patients without residual renal function. Residual kidney function (RKF) should be a better parameter for these patients but need to be calculated as the mean of creatinine and urea clearance based on urine collection performed in the whole inter-dialytic period [25]. However, the levels of pre-dialysis Scr can reflected the nutritional status [26, 27], while malnutrition was also significantly related with mortality risk among elderly hemodialysis patients [2]. Thus, SUA/Scr could reflect nutrition-normalized SUA level in these patients. Therefore, we explored the relationship between SUA/Scr and all-cause mortality among elderly hemodialysis patients in the present study.

## Material and methods

### Ethics

The present study was approved by the Ethics Committee of Taizhou Second People’s Hospital (KY2021-006). Written informed consent was obtained from each participant.

### Participants

The present retrospective, observational and single-center study observed 481 patients who received hemodialysis more than 8 hours per week at Taizhou Second People’s Hospital for at least 3 months from January 2015 to December 2019. Patients who had a history of malignancy, were less than 60 years old, and patients who combined or switched to other renal replacement therapies were excluded. Finally, 222 patients were included for the analysis.

### Clinical and laboratory data

Clinical baseline characteristics, such as age, sex, height and body weight, dialysis vintage, dialysis frequency, single-pool Kt/V (spKt/V), diabetes mellitus (DM), hypertension and comorbidities were obtained from our hospital hemodialysis database. Blood pressure was measured by trained hemodialysis nurses. Body mass index (BMI) was calculated by dividing weight (kilograms) by the square of the height (meters). SpKt/V calculation was performed with the formula of Daugirdas [28]. The laboratory data were collected before hemodialysis, including hemoglobin (Hb), albumin (ALB), total cholesterol (TC), serum uric acid (SUA), serum creatinine (Scr), calcium (Ca) and other laboratory indexes [Siemens Pipeline Biochemical Analyzer (Siemens, Inc, Munich, Germany)]. The serum uric acid to creatinine ratio was calculated by dividing serum uric acid by serum creatinine. We examined all-cause mortality from our medical record system, including cardiovascular disease (CVD) mortality and cancer during the follow-up period.

### Statistical analysis

Continuous variables conforming to a normal distribution are described by means ± standard deviations, while nonnormally distributed data are represented by medians (interquartile ranges [IQRs]). The difference between groups of normally distributed continuous variables was tested by Student’s t test. For nonnormally distributed data, the nonparametric Mann-Whitney U test was used. Chi-square (χ^2^) tests were used for the comparison of categorized variables. The Spearman correlation coefficient was used to calculate the selected variables related to SUA/Scr. Multiple Cox regression was used to analyze the association of SUA/Scr and all-cause and cardiovascular disease mortality. The analyses were preformed considering two models: Model 1: adjusted for age, gender and BMI; Model 2: adjusted for Model 1 +PA, dialysis vintage, dialysis frequency, spKt/V, diabetes mellitus, hypertension and other comorbidities. The survival rate of each group was calculated by the Kaplan-Meier method, and the ratio of survival curves was analyzed by the log-rank test. Net Reclassification Improvement (NRI) were performed to demonstrate the predictive and accuracy value of SUA, Scr and SUA/Scr. NRI was calculated as previous study reports [29]. *P*<0.05 was considered statistically significant. IBM SPSS Statistics 23.0 (SPSS, Inc., Chicago, USA) was used for data analysis.

## Results

### Demographic and baseline characteristics of elderly hemodialysis patients

A total of 222 patients were enrolled in the analysis, including 133 males and 89 females. The median age of all patients was 71.0 (65.0, 77.0) years old. During the 19-month observation period, 78 patients died.

The demographic and baseline characteristics of surviving and nonsurviving patients are shown in Table 1. Individuals in the nonsurviving group had significantly older ages than surviving patients (*P*<0.001). Furthermore, the patients who died had lower BMI (*P*=0.004), serum creatinine (*P*=0.005) and prealbumin (*P*=0.006). Other baseline characteristics did not show significant differences between groups. Apparently, patients in the nonsurviving group had a higher SUA/Scr than surviving patients (*P*=0.001).

**Table 1:** The baseline data of elderly hemodialysis patients grouped by whether death

	Normal range	Survival Group	Death Group	*P*-value
Age (years)	-	68.00 (64.25, 74.00)	74.50 (68.00, 80.00)	< 0.001***
Gender (male/female)	-	87/57	46/32	0.834
BMI (Kg/m^2^)	-	21.87 (20.26, 24.42)	20.96 (18.81, 23.33)	0.004**
SBP (mmHg)	90–140	146.62 ± 24.18	147.04 ± 28.48	0.908
DBP (mmHg)	60–90	80.00 (70.00, 88.00)	79.00 (65.00, 87.25)	0.448
Hb (g/L)	130–175	90.00 (72.00, 110.00)	91.00 (71.75, 112.50)	0.754
BUN (mmol/L)	3.1–9.5	22.85 (16.02, 31.03)	21.98 (15.82, 30.74)	0.652
Scr (umol/L)	57–111	664.52 ± 267.49	561.03 ± 238.80	0.005**
SUA (umol/L)	200–420	417.00 (322.50, 507.75)	433.50 (350.25, 545.50)	0.243
ALB (g/L)	40–55	35.65 (32.70, 39.38)	35.00 (30.83, 38.00)	0.12
PA (mg/L)	200–430	253.95 ± 86.94	215.54 ± 91.50	0.006**
TC (mmol/L)	≤ 5.2	3.66 (3.05, 4.12)	3.61 (2.98, 4.00)	0.419
TG (mmol/L)	≤ 2.25	1.09 (0.69, 1.64)	0.98 (0.70, 1.49)	0.482
Ca (mmol/L)	2.11–2.52	2.12 (1.95, 2.26)	2.12 (2.01, 2.21)	0.936
P (mmol/L)	0.85–1.51	1.76 (1.41, 2.06)	1.63 (1.31, 2.09)	0.422
SUA/Scr	**-**	0.60 (0.47, 0.88)	0.80 (0.54, 1.14)	0.001**

*Abbreviations: BMI* Body Mass Index, *SBP* Systolic Blood Pressure, *DBP* Diastolic Blood Pressure, *Hb* Hemoglobin, *BUN* Blood Urea Nitrogen, *SUA* Serum Uric Acid, *Scr* Serum Creatinine, *ALB* Albumin, *PA* Prealbumin, *TC* Total Cholesterol, *TG* triglyceride, *Ca* Calcium, *P* Phosphorus, *SUA/Scr* Serum Uric acid to Serum creatinine ratio

*Note:* *, *P* < 0.05; **, *P* < 0.01; ***, *P* < 0.001

### The correlation between SUA/Scr and selected variables

Table 2 shows that SUA/Scr was significantly positively correlated with age (*P*=0.002). The SUA/Scr was significantly negatively correlated with diastolic blood pressure (*P*=0.012), albumin (*P*<0.001), prealbumin (*P*=0.001), serum calcium (*P*=0.002) and serum phosphorus (*P*=0.037).

**Table 2:** Spearman correlation between SUA/Scr and variables

variables	Correlation coefficient	*P*-value
Age	0.202	0.002**
BMI	0.025	0.708
SBP	-0.120	0.073
DBP	-0.168	0.012*
Hb	-0.119	0.077
BUN	-0.025	0.714
ALB	-0.265	< 0.001***
PA	-0.243	0.001**
TC	0.074	0.274
TG	0.108	0.107
Ca	-0.208	0.002**
P	-0.140	0.037*

*Abbreviations: BMI* Body Mass Index, *SBP* Systolic Blood Pressure, *DBP* Diastolic Blood Pressure, *Hb* Hemoglobin, *BUN* Blood Urea rea nitrogen, *SUA* Serum Uric Acid, *Scr* Serum Creatinine, *ALB* Albumin, *PA* Prealbumin, *TC* Total Cholesterol, *TG* Triglyceride, *Ca* Calcium, *P* Phosphorus

*Note:* *, *P* < 0.05; **, *P* < 0.01; ***, *P* < 0.001

### A higher ratio of SUA/Scr is associated with a risk of all-cause mortality among elderly hemodialysis patients

**Fig. 1:**
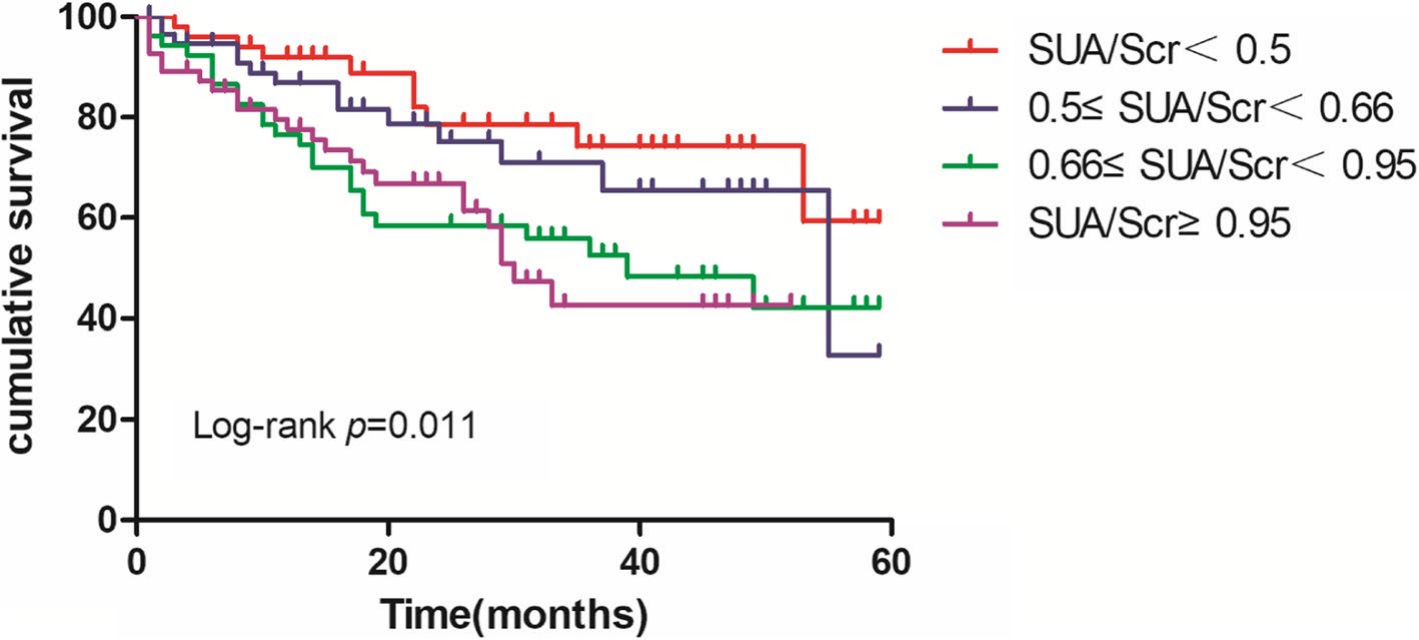
Kaplan–Meier curves for all-cause mortality among elderly hemodialysis patients grouped by SUA/Scr level

Patients were divided into four groups according to SUA/Scr quartile. The Kaplan-Meier curve was used to compare the accumulated mortality among the four groups. As shown in Figure 1, the higher SUA/Scr group had significantly higher all-cause mortality than the lower SUA/Scr group (*P*=0.011). Furthermore, we found that significant NRI was achieved when compared SUA/Scr with Scr (additive NRI=0.476, *P*<0.001) and SUA separately (additive NRI=0.214, *P*=0.015).

### SUA/Scr was an independent risk factor for all-cause mortality among elderly hemodialysis patients

Multiple Cox regression was further used to test the association of SUA/Scr and all-cause mortality. SUA/Scr was inserted in the COX regression models as a continuous variable. Consistently, as shown in Table 3, SUA/Scr was positively associated with all-cause mortality (HR=1.522 [95% CI 1.233-1.879], *P*<0.001= in the crude model. Furthermore, SUA/Scr was an independent risk factor for all-cause mortality (HR=1.292 [95% CI 1.013-1.648], *P*=0.039) after adjusting for age, sex, BMI, prealbumin, dialysis vintage, dialysis frequency, spKt/V, DM, hypertension and comorbidities. Additionally, SUA/Scr was also strongly associated with cardiovascular disease mortality (supplemental Table 1).

**Table 3:** Association of SUA/Scr with all-cause mortality by multiple Cox hazards regression analysis

	Wald	SEM	*HR*	(95%*CI*)	*P*-value
Crude Model	15.271	0.107	1.522	1.233–1.879	< 0.001***
Model 1	12.738	0.106	1.459	1.186–1.795	< 0.001***
Model 2	4.269	0.124	1.292	1.013–1.648	0.039*

SUA/Scr as a continuous variable.Model 1: adjusted for age, gender and BMI; Model 2: adjusted for age, gender, BMI, PA, dialysis vintage, dialysis frequency, spKt/V, DM, hypertension and comorbidities

*Abbreviations: BMI* Body Mass Index, *PA* Prealbumin, *DM*, Diabetes Mellitus

*Note:* *, *P* < 0.05; **, *P* < 0.01; ***, *P* < 0.001

## Discussion

The present study demonstrated that a higher serum uric acid to creatinine ratio increased all-cause mortality among elderly hemodialysis patients. To our knowledge, this is the first study to explore the relationship between SUA/Scr and all-cause mortality in elderly hemodialysis patients.

The influence of serum uric acid on the survival of hemodialysis patients is complex and paradoxical. The Framingham study was the first to indicate that serum uric acid was linked to cardiovascular outcomes among the general male population [30]. Interestingly, the same association was not shown among the female population. Previous studies also demonstrated that high serum uric acid levels predicted a high risk of death in hemodialysis patients [10, 31, 32]. In contrast, other studies showed that high uric acid levels were associated with a low risk of all-cause and cardiovascular mortality [33–35]. A large cohort study in Japan showed that uric acid levels may have a U-shaped association with all-cause mortality among the general population, which means that both low and high uric acid levels may increase mortality [36]. Consistently, a multicenter prospective cohort study in Chinese hemodialysis patients also certified a U-shaped pattern between serum uric acid level and all-cause mortality, cardiovascular disease (CVD) mortality and non-CVD mortality [37].

Serum uric acid and creatinine may be associated with the status of nutrition among hemodialysis patients. Considering the paradoxical effect of uric acid on hemodialysis patients, we sought to assess the relationship between serum uric acid and survival status in elderly hemodialysis patients using nutrition-normalized SUA (SUA/Scr). Additionally, SUA/Scr can reduce the interference of sex and renal function abnormalities [38]. Our previous studies have already revealed the relationship between SUA/Scr and renal progression [21–23]. The present study showed that a higher ratio of SUA/Scr predicted a higher risk of all-cause mortality among elderly hemodialysis patients. The link remained significant but attenuated after adjusting for other factors, such as age, sex, BMI, prealbumin, dialysis vintage, dialysis frequency, spKt/V, DM and hypertension.

The SUA/Scr was calculated using SUA and Scr. It is need to clarify whether SUA/Scr was superior to SUA or Scr on predicting all-cause mortality in elderly hemodialysis. NRI results showed that predict value on risk of all-cause mortality SUA/Scr was superior to SUA or Scr. Thus, we concluded that SUA or Scr alone was weaker than SUA/Scr as predictors of all-cause mortality among hemodialysis patients.

The present study has several limitations. First, the present study is a single-center study, which might cause selection bias. We minimize bias through quality control as far as possible. Second, the study did not distinguish the effects of residual renal function (RRF). It is attributed to the limitation of retrospective study we are unable to obtain accurate urine collection. Further multicenter studies are needed to clarify the relationship between SUA/Scr and all-cause mortality.

In summary, the present study demonstrated that the serum uric acid to creatinine ratio is strongly associated with all-cause mortality in elderly hemodialysis patients. Further verification using multicenter studies with diverse population is needed.

## Supplementary Information

**Additional file 1:** **Supplemental Table 1. **Association of SUA/Scr with cardiovascular disease mortality by multiple Cox hazards regression analysis.

## Data Availability

The data that support the findings of this study are available from Ethics Committee of Taizhou Second People’s Hospital but restrictions apply to the availability of these data, which were used under license for the current study, and so are not publicly available. Data are however available from the authors upon reasonable request and with permission of Ethics Committee of Taizhou Second People’s Hospital.

## Abbreviations

SUA/Scr: Serum uric acid to creatinine ratio
BMI: Body mass index
USRDS: United States Renal Data System
ESRD: End-stage renal disease
DOPPS: Dialysis Outcomes and Practice Patterns Study
SUA: Serum uric acid
GFR: Glomerular filtration rate
MetS: Metabolic syndrome,
NAFLD: Nonalcoholic fatty liver disease
COPD: Chronic obstructive pulmonary disease
CKD: Chronic kidney disease
Hb: Hemoglobin
ALB: Albumin
TC: Total cholesterol
Scr: Serum creatinine
Ca: Calcium
CVD: Cardiovascular disease
