# Supplementary material for: The association between the serum uric acid to creatinine ratio and all-cause mortality in elderly hemodialysis patients
Source: BMC Nephrol. 2022 May 6;23:177. doi: 10.1186/s12882-022-02798-4 (PMC9077848; doi:10.1186/s12882-022-02798-4)
Supplement: Supplementary file 1 — Additional file 1: Supplemental Table 1. Association of SUA/Scr with cardiovascular disease mortality by multiple Cox hazards regression analysis. [file 12882_2022_2798_MOESM1_ESM.docx]

Supplement data

**Supplemental Table 1** Association of SUA/Scr with cardiovascular disease mortality by multiple Cox hazards regression analysis.

|  | Wald | SEM | *HR* | (95%*CI*) | P-value |
| --- | --- | --- | --- | --- | --- |
| Crude Model | 22.992 | 0.138 | 1.934 | 1.477-2.532 | ＜0.001*** |
| Model 1 | 20.967 | 0.139 | 1.888 | 1.438-2.478 | ＜0.001*** |
| Model 2 | 8.441 | 0.175 | 1.660 | 1.179-2.337 | 0.004** |

Model 1: adjusted for age, gender and BMI;

Model 2: adjusted for age, gender, BMI, PA, dialysis vintage, dialysis frequency, spKt/V, DM, hypertension and comorbidities.

Note: *, P＜0.05；**, P＜0.01；***, P＜0.001

Abbreviations: BMI, body mass index; PA, prealbumin; DM, diabetes mellitus.
